# Looks can be deceiving: the deceptive milkcaps (*Lactifluus*, Russulaceae) exhibit low morphological variance but harbour high genetic diversity

**DOI:** 10.1186/s43008-019-0017-3

**Published:** 2019-09-18

**Authors:** Lynn Delgat, Glen Dierickx, Serge De Wilde, Claudio Angelini, Eske De Crop, Ruben De Lange, Roy Halling, Cathrin Manz, Jorinde Nuytinck, Annemieke Verbeken

**Affiliations:** 1 Department of Biology, Research group Mycology, Ghent University, K.L. Ledeganckstraat 35, 9000 Ghent, Belgium; 2 Via Cappuccini 78/8, I-33170 Pordenone, Italy; 3 National Botanical Garden of Santo Domingo (JBSD), Santo Domingo, Dominican Republic; 4 Institute of Systematic Botany, The New York Botanical Garden, 2900 Southern Blvd, Bronx, NY 10458-5126 USA; 5 Faculty of Biology, Systematic Botany and Mycology, University of Marburg, Karl-von-Frisch-Straße 8, 35043 Marburg, Germany; 6 Naturalis Biodiversity Center, P.O. Box 9517, 2300RA Leiden, The Netherlands

**Keywords:** *Basidiomycota*, *Russulales*, *Lactifluus* sect. *Albati*, Taxonomy, Phylogeny, New taxa

## Abstract

The ectomycorrhizal genus *Lactifluus* is known to contain many species complexes, consisting of morphologically very similar species, which can be considered cryptic or pseudocryptic. In this paper, a thorough molecular study is performed of the clade around *Lactifluus deceptivus* (originally described by Peck from North America) or the deceptive milkcaps. Even though most collections were identified as *L. deceptivus*, the clade is shown to contain at least 15 species, distributed across Asia and America, indicating that the *L. deceptivus* clade represents a species complex. These species are morphologically very similar and are characterized by a tomentose pileus with thin-walled hyphae and a velvety stipe with thick-walled hyphae. An ITS1 sequence was obtained through Illumina sequencing for the lectotype of *L. deceptivus*, dating from 1885, revealing which clade represents the true *L. deceptivus*. In addition, it is shown that three other described species also belong to the *L. deceptivus* clade: *L. arcuatus*, *L. caeruleitinctus* and *L. mordax,* and molecularly confirmed that *L. tomentoso-marginatus* represents a synonym of *L. deceptivus*. Furthermore, two new Neotropical species are described: *Lactifluus hallingii* and *L. domingensis*.

## INTRODUCTION

*Lactifluus* is a genus of ectomycorrhizal fungi which has its main distribution in the tropics. More than in *Lactarius*, the genus in which *Lactifluus* representatives used to be included (Buyck et al. 2008), it is known for its high molecular diversity, with many species complexes occurring throughout the genus (Stubbe 2012; Van de Putte 2012; De Crop 2016). These species complexes consist of several closely related species, with limited morphological variability, making them hard to distinguish from each other.

A good example of such species complexes can be found in *Lactifluus* section *Albati*. Species in this section are characterized by large white basidiocarps, a velutinous cap, an acrid taste of the context, the presence of macrocystidia and a pileipellis consisting of hyphae, without isodiametric cells. This section was first thought to contain only temperate representatives, with species known from Europe and North America. More recently, some Asian species belonging to this section were discovered, expanding its known distribution across the Northern hemisphere (Wen and Ying 2005; Le et al. 2007b).

The section contains two distinct clades: one with *L. vellereus*, and another with *L. deceptivus*. The *L. vellereus* clade contains the European *L. vellereus* and *L. bertillonii*, the North American *L. subvellereus* and the Asian *L. pilosus* and *L. puberulus,* in addition to a myriad of names at the variety level. In contrast, *L. deceptivus* is described from North America. The also North American *L. tomentoso-marginatus* was synonymized with *L. deceptivus* based on morphological study of the type specimens (Montoya and Bandala 2005). However, molecular data suggested that specimens identified as *L. deceptivus* represented several species, and a detailed molecular study is imperative to resolve the species boundaries in this complex. Therefore, a phylogeny based on multiple loci of the *L. deceptivus* clade was constructed in this paper, including samples from different biogeographic regions.

## MATERIAL AND METHODS

### Sampling

Samples were included from sampling expeditions to the United States of America (2005), Canada (2007), Vietnam (2011), Dominican Republic (2016–2017) and Panama (2018). In addition, loans were requested from the NY, NYS, FLAS and MICH herbaria. Type specimens of described species that (could) belong to *L.* sect. *Albati* were requested, but of these the type of *L. caeruleitinctus* was in too poor condition for sequencing. For *L. deceptivus*, no type was designated in the original description. However, Hesler and Smith (1979) indirectly designated a lectotype, and this collection, in addition to five other collections mentioned in the studied material were requested.

### Morphological analysis

Macroscopic characters were observed from fresh material with colour codes referring to Kornerup and Wanscher (1978). Microscopic characters were observed from dried material. Basidiospores were mounted in Melzer’s reagent and hymenium, pileipellis and stipitipellis were studied in Congo red in L4. The basidiospore measurements (i.e. length, width and Q = quotient of length and width) are given as [Av_a_-2 × SD_a_]–*Av*_*a*_*–Av*_*b*_–[Av_b_ + 2 × SD_b_], in which *Av*_*a*_ = lowest mean value for the measured specimens, *Av*_*b*_ = greatest mean value, SD_a/b_ = standard deviation of the measurements with the lowest and greatest mean value respectively (number of spores measured per specimen = 20). Basidiospores were measured in side view without ornamentation. Measurements of basidia, cystidia and marginal cells are given as [Av-2 × SD]–*Av*–[Av + 2 × SD], based on minimum 47 measurements per species. Measurements of basidia do not include sterigmata. Line drawings of spores were made based on stacked photographs (Nikon Eclipse Ni-U, stacking software: Extended Depth of Field, Nikon Nis Elements module) 5000× magnified, those of other elements and sections were made with the aid of a drawing tube at magnification 1500× (Olympus cx31 microscope).

### Molecular analysis

DNA was extracted from fresh material preserved in CTAB (Cetyl trimethylammonium bromide) using the CTAB extraction described in Nuytinck and Verbeken (2003). A modified CTAB protocol (Tel-Zur et al. 1999; mod. by Agentschap Plantentuin Meise) was used for dried collections. PCR amplification protocols follow Le et al. (2007a). Two nuclear markers were amplified: (1) the internal transcribed spacer region of ribosomal DNA (ITS), comprising the ITS1 and ITS2 spacer regions and the ribosomal gene 5.8S, using primers ITS1F and ITS4, in addition to primers ITS5, ITS2 and 58SF for problematic material (White et al. 1990; Gardes and Bruns 1993; Tedersoo et al. 2013), and (2) the region between the conserved domains 6 and 7 of the second largest subunit of the RNA polymerase II (*RPB2*), using primers bRPB2-6F and fRPB2-7cR (Liu et al. 1999; Matheny 2005).

PCR products were sequenced using an automated ABI 3730 XL capillary sequencer (Life Technology) at Macrogen. Forward and reverse sequences were assembled into contigs and edited with Sequencher v5.0 (Gene Codes Corporation, Ann Arbor, MI, U.S.A.) or BioloMICS (BioAware SA NV).

For the 134-year-old type specimen of *L. deceptivus,* Illumina MiSeq sequencing was chosen as an alternative to conventional Sanger sequencing to overcome the high fragmentation of the ancient DNA and the occurrence of non-target DNA from fungal contaminants. Also, type specimens of *L. mordax* and *L. tomentoso-marginatus* were sequenced with this method. Amplification of the ITS1 region was carried out using a two-step PCR process. In the first PCR, universal ITS1-F/ITS2 primers extended with Nextera™ tails (Illumina) were used following the same settings as detailed in Le et al. (2007a). After a DNA quantity and quality check, the PCR product was cleaned up with the NucleoMag NGS Clean-up and Size Select kit (Machery-Nagel). In the second PCR, a Nextera™ XT label (Illumina) was added to the amplicon under the following conditions: 3 μL of template DNA, 1 μL of each primer (10 pmol/μL), and 15 μL of Master Mix for a final volume of 20 μL. Amplification conditions were: 95 °C for 10 min, 8 cycles of 30 s at 95 °C, 60 s at 55 °C and 30 s at 72 °C, followed by 7 min at 72 °C. After quantification and clean-up, the sample was sent to BaseClear (Leiden, the Netherlands) for paired-end sequencing using the Illumina MiSeq technology (2 × 300 bp) amongst a batch of other amplicons with different Nextera™ labels.

The Illumina sequence reads were processed using the Naturalis Galaxy v.19.01 instance. The reads were demultiplexed on their unique tag to isolate the reads from specific specimens. R1 and R2 reads from paired-end sequencing were merged with FLASH (Magoc and Salzberg, 2011) with the minimum overlap size set at 100 bp. Reads shorter than 250 bp or with more than 8 consecutive N’s or a Phred score lower than 28 were discarded and primers were trimmed with Cutadapt (Martin 2011). After a quality control step with PRINSEQ (Schmieder and Edwards 2011), the sequences were dereplicated, sorted by size and clustered in zero-radius OTU’s with the UNOISE algorithm (Edgar and Flyvbjerg 2015; Edgar 2016) to denoise the amplicon reads. Chimera sequences were removed with the VSEARCH UCHIME algorithm (Edgar et al. 2011). Each zero-radius OTU was then taxonomically assigned by using a BLASTN search (Altschul et al. 1997) against the UNITE and GenBank databases. An OTU abundance table was created and combined with the taxonomic assignments.

The dataset was supplemented with closely related sequences retrieved from GenBank and worldwide reference sequences from De Crop et al. (2017) (Table 1). Metadata of collections in the *L. deceptivus* complex are given in Table 2. Sequences were aligned online in the multiple sequence alignment program MAFFT v7 (Katoh and Toh 2008), using the E-INS-I strategy. Trailing ends were trimmed and the alignment was manually edited where needed in Mega 6 (Tamura et al. 2013). The ITS+LSU alignment was partitioned into partial 18S, ITS1, 5.8S, ITS2 and partial 28S. The *RPB2* alignment was partitioned into the intron and the first, second and third codon positions of the exon. PartitionFinder was used to find the appropriate partitioning scheme (Lanfear et al. 2017). Maximum likelihood (ML) analyses, using RAxML v8.0.24 (Stamatakis 2014), were combined with the Rapid Bootstrapping algorithm with 1000 replicates under the GTRCAT option (Stamatakis et al. 2008). There was no supported conflict between the separate gene trees, so they were concatenated. The concatenated alignment can be obtained from the first author or TreeBASE (ID 24889). All analyses were conducted on the CIPRES Science Gateway (Miller et al. 2010).

**Table 1:** Specimens and GenBank accession numbers of DNA sequences used in the molecular analyses

Species	Number	Fungarium	ITS	RPB2
*Amylostereum laevigatum*	CBS 623.84	CBS	AY781246	AY218469
*Auriscalpium vulgare*	PBM 944	WTU	DQ911613	AY218472
*Bondarzewia montana*	AFTOL 452	DAOM	DQ200923	AY218474
*Echinodontium tinctorium*	AFTOL 455	DAOM	AY854088	AY218482
*Heterobasidion annosum*	AFTOL 470	DAOM	DQ206988	AH013701
*Lactarius fuliginosus*	MTB 97–24	GENT	JQ446111	JQ446240
*Lactarius hatsudake*	FH 12–052	GENT	KR364085	KR364285
*Lactarius leoninus*	DS 07–454	GENT	KF220055	JN375592
*Lactarius miniatescens*	AV 11–177	GENT	KR364059	KR364315
*Lactarius olympianus*	ED 08–018	GENT	KR364089	KR364320
*Lactarius pseudodeceptivus*	Smith 71,932	MICH	MK931348	–
*Lactarius pseudodeceptivus*	Smith 29,178	MICH	MK931349	–
*Lactarius pseudodeceptivus*	Smith 89,282	MICH	MK931350	–
*Lactarius scrobiculatus*	JN 2001–058	GENT	KF432968	KR364344
*Lactarius tenellus*	ADK 3598	GENT	KF133280	KF133345
*Lactifluus acrissimus*	EDC 11–112	GENT	KR364041	KR364254
*Lactifluus allardii*	JN 2004–008	GENT	KF220016	KF220217
*Lactifluus arcuatus*	FLAS-F-16366	FLAS	MK931344	–
*Lactifluus arcuatus*	FLAS-F-60197	FLAS	MF153025	MK937138
*Lactifluus aurantiifolius*	AV 94–063	GENT	KR364017	–
*Lactifluus bertillonii*	JN 2012–016	GENT	KR364087	KR364261
*Lactifluus brachystegiae*	AV 99–002	GENT	KR364018	KR364262
*Lactifluus caeruleitinctus*	FLAS-F-59238	FLAS	MK931345	–
*Lactifluus clarkeae*	MN 2004002	L	KR364011	KR364268
*Lactifluus cocosmus*	ADK 4462	GENT	KR364013	KR364269
*Lactifluus deceptivus*	AV 05–275	GENT	MK931336	–
*Lactifluus deceptivus*	Smith 84,511	MICH	MK931351	–
*Lactifluus deceptivus*	PC BB2004–259	PC	EU598200	–
*Lactifluus deceptivus*	NYS-F-000959	NYS	MN251093	–
*Lactifluus densifolius*	AV 11–111	GENT	KR364057	KR364273
*Lactifluus domingensis*	ANGE542	JBSD	MK931339	MK937130
*Lactifluus domingensis*	ANGE1035	JBSD	MK931340	MK937131
*Lactifluus domingensis*	ANGE838	JBSD	MK931341	MK937132
*Lactifluus domingensis*	ANGE837	JBSD	MK931342	MK937133
*Lactifluus edulis*	FN 05–628	GENT	KR364020	KR364275
*Lactifluus foetens*	ADK 3688	MEISE	KR364022	KR364278
*Lactifluus gerardii*	AV 05–375	GENT	GU258254	GU258353
*Lactifluus gymnocarpus*	EDC 12–047	GENT	KR364065	KR364282
*Lactifluus hallingii*	FH 18–077	GENT	MK931338	MK937129
*Lactifluus hallingii*	A. E. Franco-Molano 523	NY	MK931330	–
*Lactifluus hallingii*	A. E. Franco-Molano 555	NY	MK931331	–
*Lactifluus hallingii*	A. E. Franco-Molano 756	NY	MK931332	–
*Lactifluus hallingii*	R. E. Halling 4977	NY	MK931343	–
*Lactifluus hallingii*	R. E. Halling 7938	NY	MK931327	MK937127
*Lactifluus hallingii*	R. E. Halling 7993	NY	MK931333	MK937128
*Lactifluus hallingii*	NVE 520	ANDES	KF937338	–
*Lactifluus hallingii*	NVE 401	ANDES	KF937337	–
*Lactifluus hallingii*	NVE 538	ANDES	KF937339	–
*Lactifluus heimii*	EDC 11–082	GENT	KR364040	KR364286
*Lactifluus luteolus*	AV 05–253	GENT	KR364016	KJ210067
*Lactifluus madagascariensis*	BB 99–409	PC	AY606977	DQ421914
*Lactifluus mordax*	FLAS-F-52759	FLAS	MK931346	–
*Lactifluus mordax*	FLAS-F-61658	FLAS	MH212033	MK937139
*Lactifluus mordax*	HDT 1570	MICH	MN251096	–
*Lactifluus multiceps*	TH 9154A	BRG, DUKE	JN168731	–
*Lactifluus oedematopus*	KVP 12–001	GENT	KR364100	KR364319
*Lactifluus pegleri*	PAM-Mart12–91	LIP	KP691416	KP691433
*Lactifluus phlebonemus*	EDC 12–023	GENT	KR364062	KR364322
*Lactifluus pilosus*	LTH 205	GENT	KR364006	KR364323
*Lactifluus Piperati*	HKAS J7008	HKAS	KR364108	KR364309
*Lactifluus piperatus*	2001 08 19 68	GENT	KF220119	KF241842
*Lactifluus pseudogymnocarpus*	AV 05–085	GENT	KR364012	KR364329
*Lactifluus putidus*	PAM/Mart 11–013	LIP	KP691413	KP691431
*Lactifluus ramipilosus*	EDC 14–503	GENT	KR364128	–
*Lactifluus robustus*	JN 2011–074	GENT	KR364047	KR364358
*Lactifluus rufomarginatus*	ADK 3011	GENT	KR364034	KR364336
*Lactifluus ruvubuensis*	JD 303	MEISE	KR364009	KR364310
*Lactifluus* sp.	PBM 2462 (AFTOL-ID 682)	no data	AY854089	AY803749
*Lactifluus* sp.	SDM 037	BR	KR364028	KR364291
*Lactifluus* sp.	FLAS-F-15973	FLAS	MK931347	–
*Lactifluus* sp.	FLAS-F-61618	FLAS	MH212001	MK937135
*Lactifluus* sp.	FLAS-F-61044	FLAS	MH211710	MK937136
*Lactifluus* sp.	FLAS-F-61657	FLAS	MH212032	MK937137
*Lactifluus* sp.	AV 99–012	GENT	KR364021	KR364276
*Lactifluus* sp.	AV 05–249	GENT	MK931325	MK937125
*Lactifluus* sp.	JN 2007–012	GENT	MK931326	MK937134
*Lactifluus* sp.	AV 04–181	GENT	MK931328	DQ421935
*Lactifluus* sp.	EDC 12–040	GENT	KR364063	KR364289
*Lactifluus* sp.	EDC 12–195	GENT	KR364071	KR364301
*Lactifluus* sp.	JD 907	GENT	KR364076	KR364302
*Lactifluus* sp.	JN 2011–071	GENT	KR364043	KR364255
*Lactifluus* sp.	JN 2011–077	GENT	KR364044	KR364256
*Lactifluus* sp.	AV 05–325	GENT	MK931329	–
*Lactifluus* sp.	AV 05–332	GENT	MK931334	–
*Lactifluus* sp.	AV 05–350	GENT	MK931335	MK937126
*Lactifluus* sp.	RC/Guy 09-004bis	LIP	KJ786643	KP691427
*Lactifluus* sp.	Schaffer 5895	MICH	MK931352	–
*Lactifluus* sp.	Weber 4277	MICH	MK931353	–
*Lactifluus* sp.	Ammirati 2392	MICH	MK931354	–
*Lactifluus* sp.	Ruth Mc Vaugh 1292	MICH	MK931355	–
*Lactifluus* sp.	G3264	PC	KJ786706	KP691435
*Lactifluus* sp.	TENN 065854	TENN	KR364101	KR364271
*Lactifluus* sp.	NVE 396	ANDES	KF937340	–
*Lactifluus* sp.	ASM 13521	EIU	MK931337	–
*Lactifluus* sp.	2836	No data	KJ705226	–
*Lactifluus* sp.	2225-QFB-25948	No data	KJ705225	–
*Lactifluus subkigomaensis*	EDC 11–159	GENT	KR364050	KR364295
*Lactifluus subvellereus*	AV 05–210	GENT	KR364010	KR364347
*Lactifluus tomentoso-marginatus*	MICH 11224	MICH	MN251094	–
*Lactifluus tomentoso-marginatus*	MICH 37937	MICH	MN251095	–
*Lactifluus urens*	EDC 14–032	GENT	KR364124	KR364353
*Lactifluus vellereus*	ATHU-M 8077	ATHU-M	KR364106	KR364354
*Lactifluus venezuelanus*	RC/Guad11–017	LIP	KP691411	KP691429
*Lactifluus volemoides*	MH 201187	GENT	KR364098	KR364363
*Lactifluus xerampelinus*	MH 201176	GENT	KR364099	KR364364
*Multifurca furcata*	R. E. Halling 7804	NY	DQ421995	DQ421928
*Multifurca ochricompacta*	BB 02–107	PC	DQ421984	DQ421940
*Multifurca* sp.	xp2–20,120,922-01	GENT	KR364125	–
*Multifurca stenophylla*	JET956	MEL	JX266631	–
*Multifurca zonaria*	FH 12–009	GENT	KR364083	KR364365
*Russula chloroides/delica*	FH 12–272	GENT	KF432955	KR364340
*Russula cyanoxantha*	FH 12–201	GENT	KR364093	KR364341
*Russula gracillima*	FH 12–264	GENT	KR364094	KR364342
*Russula khanchanjungae*	AV-KD-KVP 09–106	GENT	KR364129	JN375607
*Russula* sp.	EDC 12–061	GENT	KR364072	KR364338
*Russula* sp.	EDC 12–063	GENT	KR364073	KR364339
*Stereum hirsutum*	AFTOL 492	No data	AY854063	AY218520
*Vararia abortiphysa*	CBS 630.81	CBS	KR364005	KR364266

**Table 2:** Metadata of collections in the *L. deceptivus* complex

Number	Collection date	Country	Locality	Collector
A. E. Franco-Molano 523	1990	Colombia	Antioquia, Mun. Santa Rosa de Osos, Vereda El Chaquiro, finca La Española	A. E. Franco-Molano
A. E. Franco-Molano 555	1991	Colombia	Antioquia, Mun. San Pedro, Vereda La Pulgarina	A. E. Franco-Molano
A. E. Franco-Molano 756	1991	Colombia	Antioquia, Mun. Santa Rosa de Osos, Corregimiento de Aragón, vereda El Quince. Finca San Bernardo.	A. E. Franco-Molano
Ammirati 2392	22/08/1968	United States	Michigan, Marquette, Yellow Dog Pine Plains	J. F. Ammirati
ANGE1035	18/12/2017	Dominican Republic	Jarabacoa	C. Angelini
ANGE542	10/01/2016	Dominican Republic	Jarabacoa	C. Angelini
ANGE837	07/12/2016	Dominican Republic	Jarabacoa	C. Angelini
ANGE838	08/12/2016	Dominican Republic	Jarabacoa	C. Angelini
ASM 13521	13/08/2011	United States	New York, Black Pond, Adirondack Park, Franklin County	A. S. Methven
AV 04–181	13/07/2004	United States	Tennessee, Sevier County, Cascade Trail in the Greenbrier section of the Great Smokey Mountains	A. Verbeken
AV 05–249	12/08/2005	United States	North Carolina, Cataloochee, Caldwell Fork Trail	A. Verbeken
AV 05–275	12/08/2005	United States	North Carolina, Cataloochee, Rough Fork Trail	A. Verbeken
AV 05–325	15/08/2005	United States	North Carolina, Swain County, Round Bottom, Beech gap Trail	A. Verbeken
AV 05–332	15/08/2005	United States	North Carolina, Swain County, Heintoogard	A. Verbeken
AV 05–350	17/08/2005	United States	North Carolina, Swain County, Kephart Prong Trail	A. Verbeken
FH 18–077	19/06/2018	Panama	Cerro Punta, Montana Azul, Parque Internacional la Amistad	F. Hampe & C. Manz
FLAS-F-15973	13/08/1937	United States	Florida, Alachua, Gainesville	W. A. Murrill
FLAS-F-16366	15/06/1938	United States	Florida, Alachua, Gainesville	W. A. Murrill
FLAS-F-52759	25/08/1981	United States	Florida, Alachua, Gainesville, Newnan’s Lake area, west shore	Benny & Kimbrough
FLAS-F-59238	04/12/2004	United States	Florida, Okaloosa, Elgin Air Force Base	D. P. Lewis
FLAS-F-60197	24/10/2016	United States	Florida, Putnam, Ordway-Swisher Biological Station. Between Lake Rowan and Lake Barco, near Road B17 intersection	Smith Lab
FLAS-F-61044	28/06/2017	United States	Florida, Putnam, Ordway-Swisher Biological Station, northwest of Ashley Lake	D. Borland & B. Kaminsky
FLAS-F-61618	16/09/2017	United States	Florida, Alachua, Owen-Illinois Park, Windsor, Florida, USA.	B. Kaminsky
FLAS-F-61657	20/09/2017	United States	Florida, Putnam, Ordway-Swisher Biological Station, by D10 road intersection	D. Borland & B. Kaminsky
FLAS-F-61658	20/09/2017	United States	Florida, Putnam, Ordway-Swisher Biological Station, by D10 road intersection	D. Borland & B. Kaminsky
HDT 1570	22/05/1952	United States	Texas, Grimes, Navasota	H. D. Thiers
JN 2007–012	26/09/2007	Canada	Newfoundland, Avalon Peninsula, Salmonier road (90), Salmonier National Park	J. Nuytinck
JN 2011–071	16/06/2011	Viet Nam	Bi Dup Nui Ba National Park, Huyen Lac Duong, Dalat city, near Tram Kiem Lam Giang Ly	J. Nuytinck
JN 2011–077	16/06/2011	Viet Nam	Bi Dup Nui Ba National Park, Huyen Lac Duong, Dalat city, near Tram Kiem Lam Giang Ly	J. Nuytinck
MICH 11224	27/08/1973	United States	Michigan, Oscoda, Perry Creek, Mio	C. Nimke
MICH 37937	22/09/1975	United States	Michigan, Washtenaw, Winnewana Lake	A. H. Smith
NYS-F-000959	August	United States	New York, Rensselaer, Sandlake	C. H. Peck
R. E. Halling 4977	1986	Colombia	Antioquia, Mun. Santa Rosa de Osos, Near Llanos de Cuiva	R. E. Halling
R. E. Halling 7938	26/06/2000	Costa Rica	San José: Canton Dota, San Gerardo. Albergue de la Montaña, Savegre, 5 km SW of Cerro de la Muerte	R. E. Halling
R. E. Halling 7993	07/08/2000	Costa Rica	San José: Canton Dota, Jardin, 3,5 km W of Empalme	R. E. Halling
Ruth Mc Vaugh 1292	21/09/1967	Mexico	Oaxaca, 3–5 km E of Ixtlan de Juarez, along road to Capulalpan	R.B. McVaugh
Schaffer 5895	17/08/1967	Canada	Quebec, Charlevoix, Baie Saint Paul	R. L. Shaffer
Smith 84,511	22/08/1973	United States	Michigan	A. H. Smith
TENN 065854	12/08/2011	United States	New York, Paul Smith’s Franklin, Boreal Life trail, Walk No.8: Barnum Brook, New England Mycological Foray	S. Rock
Weber 4277	14/09/1974	United States	Wisconsin	N. S. Weber

## RESULTS

Illumina Miseq sequencing was used to sequence the type specimen of *L. deceptivus* collected in 1885. After library sequencing, merging read pairs and quality control steps for this type specimen, 727 ITS1 sequences were retained that were clustered in 10 zero-radius OTU’s. Only one of these belonged to the genus *Lactifluus*, and this sequence was used in further phylogenetic analysis. Other OTU’s represent contaminants, the most abundant being *Penicillium*. For the type specimens of *L. mordax* and *L. tomentoso-marginatus,* 125–271 sequences were retained, clustered in 4–6 zero-radius OTU’s, which each contained one sequence related to *L. deceptivus s. lat*.

**Fig. 1:**
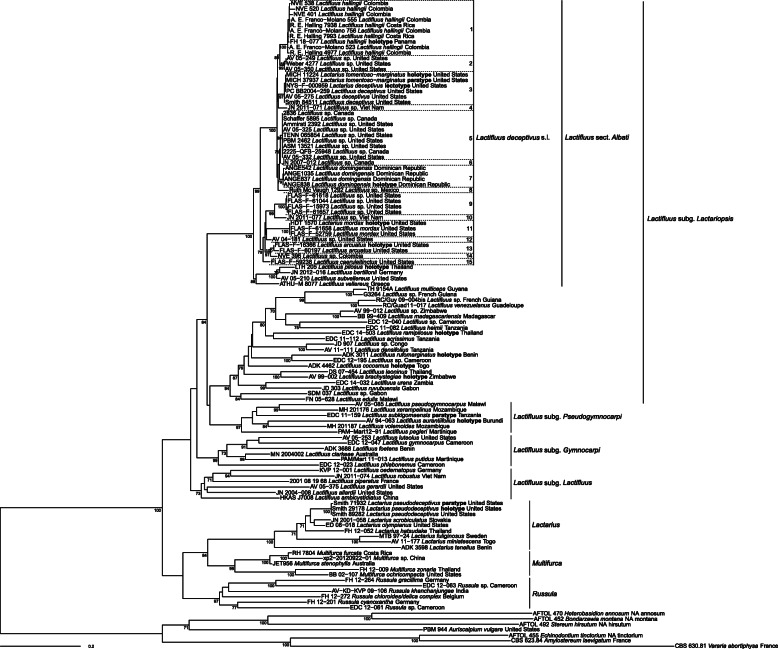
Maximum Likelihood tree based on concatenated ITS and RPB2 sequence data. Maximum Likelihood bootstrap values > 70 are shown

In total, sequences were obtained for 47 collections belonging to the *L. deceptivus* complex. Of these collections, 36 had been identified based on morphology as *L. deceptivus* (most other collections were not identified to species level). The phylogeny shows that these collections represent at least 15 species (Fig. 1). Most species originate from the Nearctic, but also two Indomalayan and three Neotropical species were found. Only four species were described thusfar, so an additional 11 new species were uncovered by the molecular analysis.

## TAXONOMY

Genus *Lactifluus* (Pers.) Roussel

*Lactifluus* subgenus *Lactariopsis* (Henn.) Verbeken

*Lactifluus* section *Albati* (Bataille) Verbeken

**Lactifluus domingensis** Delgat & Angelini, **sp. nov.**

MycoBank MB831084

(Figs. 2 and 3)

**Fig. 2:**
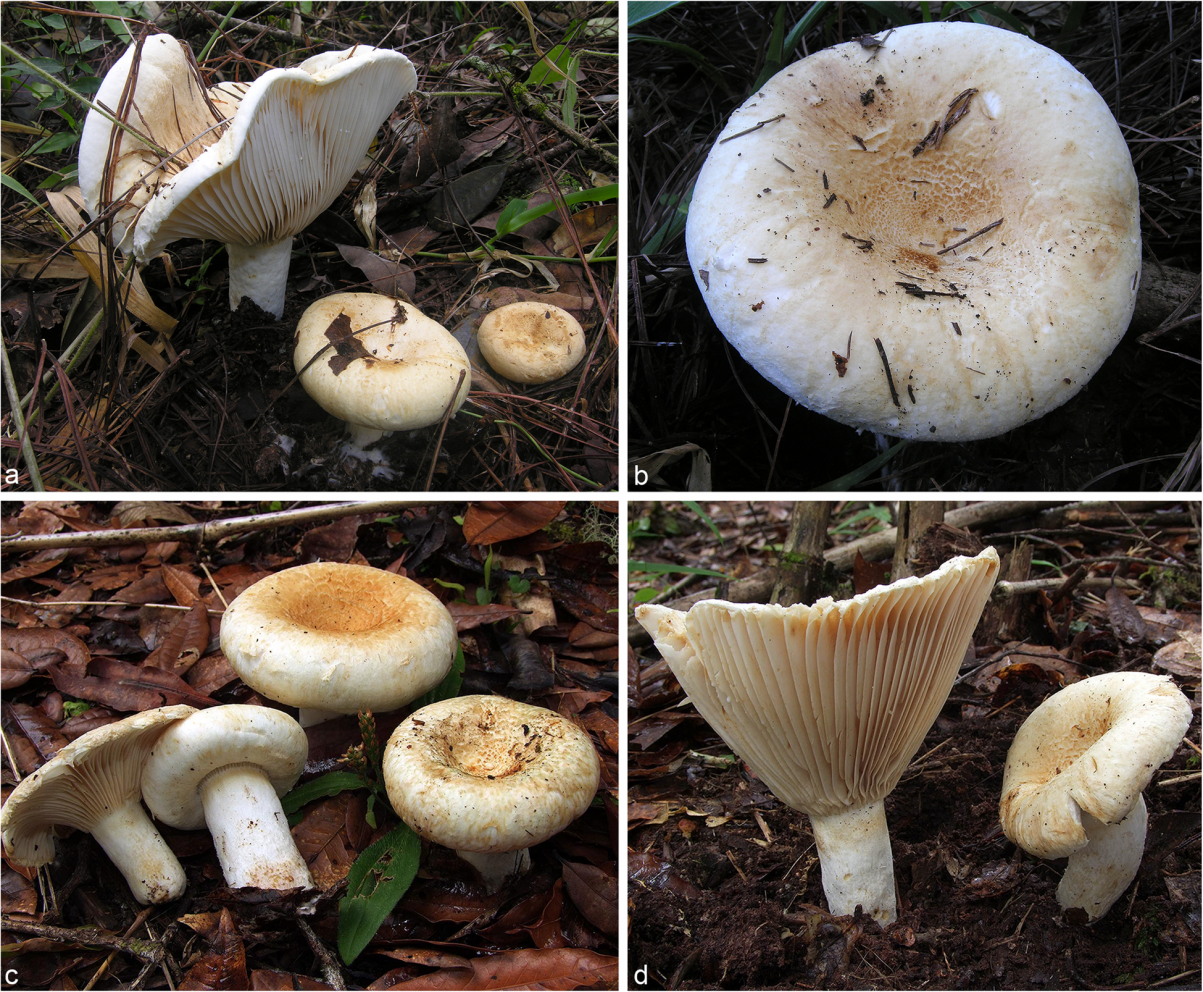
Basidiomes. **a** *Lactifluus domingensis* (ANGE838–holotype). **b** *Lactifluus domingensis* (ANGE837). **c**–**d** *Lactifluus hallingii* (FH 18–077–holotype)

**Fig. 3:**
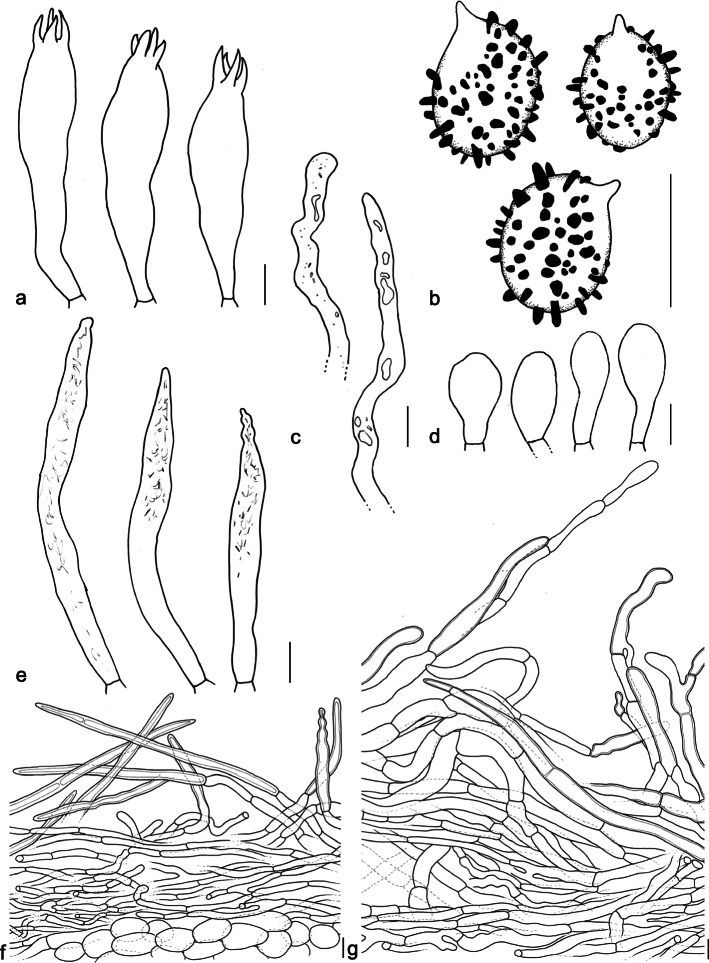
*Lactifluus domingensis* (ANGE 542, ANGE 837, ANGE838). **a** Basidia. **b** Basidiospores. **c** Pseudocystidia. **d** Marginal cells. **e** Macrocystidia. **f** Section through the stipitipellis. **g** Section through the pileipellis. Bars = 10 μm

*Etymology*: Refers to the island where the species was found. (Hispaniola previously consisted of Santo Domingo and Saint-Domingue, currently Dominican Republic and Haiti respectively.)

*Diagnosis*: Differs from clade 5 in the slightly lower average Q of the basidiospores, the slightly larger basidia, the slightly longer cystidia, the Hispaniolan distribution and the association with *Pinus occidentalis*.

*Type*: **Dominican Republic**: *La Vega province*: Jarabacoa, mixed wood mostly with *Pinus occidentalis*, 8 Dec.2016, *C. Angelini ANGE838* (JBSD 130755 – holotype JBSD; GENT – isoptype).

*Description*: *Pileus* 35–100 mm diam, compact, at first convex and umbilicate, becoming flat, with depressed centre that becomes deeply depressed with maturity; surface at first bald, but soon roughening, becoming torn into patches or scales, dry to sticky at the centre; margin at first involute and clothed with a soft or cottony tomentum, then spreading or becoming elevated and more or less fibrillose, with a hand-lens loosely arranged brown pigmented hairs are visible; surface whitish at first, soon discolouring tan and brownish. *Stipe* 17–50 × 10–20 mm, rather short (compared to the diameter of the cap), usually tapering to base; surface dry, irregularly rugged, slightly velvety towards the base, whitish, discolouring brownish or tan; context solid. *Lamellae* adnate or slightly decurrent, with lamellulae of various length, rather broad, to 5 mm wide, subdistant, occasionally forking, whitish or pale yellowish, becoming cream coloured and finally dingy tan, usually staining and discolouring brownish; edge entire and concolourous. *Context* firm, white, unchanging when cut; smell pungent at maturity; taste strongly acrid. Latex white; unchanging; often staining brownish. *Chemical reactions*: Cap surface reddish with KOH.

*Basidiospores* broadly ellipsoid to ellipsoid, 8–*8.9–10.1*–11.5 × 6.3–*7–7.7*–8.7 μm (Q = 1.15–*1.25–1.31*–1.50), ornamentation to 1.7 μm high, consisting of isolated conical warts; plage inamyloid, rarely centrally weakly amyloid. *Basidia* (42–)43–*53*–63.5 × 9.5–*12.5*–15.5 μm, subclavate, majority 4-spored, some 2- or 1-spored. *Pleuromacrocystidia* 35–*62.5*–90(− 101) × 5–*7*–9 μm, abundant, cylindrical with tapering, mucronate or moniliform apex, sometimes branching, thin-walled. *Pleuropseudocystidia* 3.5–9 μm diam, not to slightly emergent. *Sterile elements* 27–*37*–46.5 × 4–*5*–6.5 μm, cylindrical, septate, with rounded apex. *Lamellar edge* sterile; marginal cells 9.5–*23.5*–38(40) × 4.5–*9.5*–14(− 16) μm, cylindrical to clavate, sometimes septate. *Hymenophoral trama* mixed, with hyphae, lactifers and sphaerocytes. *Pileipellis* a very loosely arranged cutis to trichoderm, up to 200 μm thick, composed of very broad (8–20, sometimes 30 μm diam.) and slightly thick-walled hairs, that are periclinally to anticlinally arranged and have a pale brown pigmentation. Some branchings and bulges of the hyphae are present. *Stipitipellis* a loosely arranged lamprotrichoderm on top of a layer of globose cells, up to 150 μm thick; terminal elements thick-walled, 50–150 × 5–8 μm, usually straight and hair-shaped and with tapering apex, periclinally to anticlinally arranged.

*Ecology*: Found in montane forests with *Pinus occidentalis.*

*Distribution*: Only known from the Dominican Republic, on the island of Hispaniola.

*Remarks*: Several hosts have been reported for *Lactifluus deceptivus s. lat*., such as *Pinus*, *Tsuga*, and *Quercus* species. In the Dominican Republic, where this new species was found, no *Fagaceae* occur. There are two species of pines on the island: the endemic *Pinus occidentalis* (in the central Cordillera) and the introduced *P. caribaea* (in the northern Cordillera). *Lactifluus domingensis* has been found exclusively with the endemic *P. occidentalis* and never with the introduced *P. caribaea*. This association with *P. occidentalis*, and therefore Hispaniolan distribution, is probably a unique feature of the species. So far, no other *Lactifluus* species are known from this island. Aside from its distinctive distribution, *L. domingensis* bears great resemblance to the other species of the *Lactifluus deceptivus* species complex, both macro- and microscopically. The phylogeny shows that *L. domingensis* is most closely related to a single collection from Canada (clade 6) and a clade with a northern North American distribution: clade 5 (Fig. 1) (although unsupported, but strongly supported in the separate ITS phylogeny). Clade 5 differs from *L. domingensis* by the slightly higher average Q (1.22–*1.34–1.35*–1.47), the slightly smaller basidia (24–*43*–62 × 3.5–*11.5*–19 μm) and the slightly shorter cystidia (44–*54*–63.5 × 4–*7.5*–10.5 μm). Compared to the two described species from Florida, *L. arcuatus* has distinctly smaller spores (4–6 μm long), and *L. caeruleitinctus* has blue tinges in the stipe which are lacking in *L. domingensis*. Lastly, *L. hallingii* also has a Central American distribution, but this species was found with *Quercus* species. In addition, there are some subtle microscopic differences: *L. hallingii* has slightly lower spore ornamentation (up to 1.5 μm), somewhat longer basidia (45.5–*63.5*–81.5(− 83) × 10–*13*–16 μm), somewhat differently shaped macrocystidia (i.e. more rarely a mucronate or moniliform apex, more often with a rounded or tapering apex) and an irregular cutis as a pileipellis.

*Other specimens examined*: **Dominican Republic**: *La Vega province*: Jarabacoa: mixed woods mostly with *Pinus occidentalis*, on soil, 10 Jan./2016, *C. Angelini ANGE542* (JBSD 130756); ibid., 7 Dec. 2016, *C. Angelini ANGE837* (JBSD 130757); ibid., 18 Dec. 2017, *C. Angelini* ANGE1035 (JBSD 130758).

**Lactifluus hallingii** Delgat & De Wilde, **sp. nov.**

MycoBank MB831085

(Figs. 2 and 4)

**Fig. 4:**
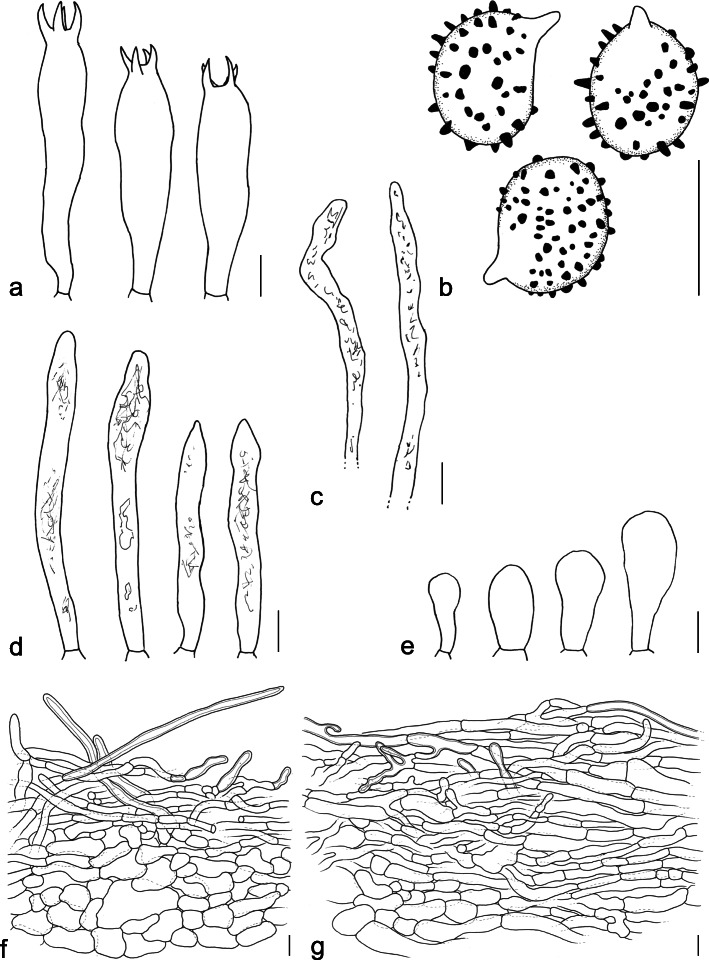
*Lactifluus hallingii* (AEF 555, AEF 756, FH 18–077, REH 7993). **a** Basidia. **b** Basidiospores. **c** Pseudocystidia. **d** Macrocystidia. **e** Marginal cells. **f** Section through the stipitipellis. **g** Section through the pileipellis. Bars = 10 μm

*Etymology*: Refers to mycologist Roy E. Halling, who made several collections of this species.

*Diagnosis*: Differs from clade 2 in the larger basidia, the longer macrocystidia and the Neotropical distribution.

*Type*: **Panama:** Cerro Punta, Montana Azul, Parque Internacional la Amistad (8.894361°; − 82.581956°, alt. 2338 m), soil with *Quercus* sp., 19 June 2018, *F. Hampe* & *C. Manz FH 18–077* (GENT – holotype; UCH 10505 – isotype)

*Description*: *Pileus* 40–165(− 240) mm diam, infundibuliform; margin strongly involute when young; surface dry, matted tomentose at first, eventually fibrillose to squamulose at disc, with cottony roll of tissue at margin when young, later appressed fibrillose to tomentose, cream or pale orange (4A2–4A5, 5A3) at first, then browner near brownish orange (6C6) at disc and paler (whitish) towards the margin. Stipe 30–90 × 11–35 mm, cylindrical, sometimes curved; surface white, staining brownish (4A3–4) where injured, dry, tomentose to pubescent. *Lamellae* adnate to subdecurrent to decurrent, up to 6 mm wide, subdistant, occasionally forking, white to cream (4A2–3); edge entire and concolourous. *Context* firm, white, unchanging when cut; smell fruity and cheesy; taste very acrid. *Latex* scarce to abundant, white, staining tissues pinkish to eventually brownish; taste very acrid. *Chemical reactions*: context green blue with guaiac, orange with FeSO_4_; cap surface reddish cinnamon with KOH.

*Basidiospores* broadly ellipsoid to ellipsoid, 8.1–*9.6–10.4*–11.3 × 6.7–*7.4–8.5*–9.5 μm (Q = 1.15–*1.21–1.32*–1.47); ornamentation up to 1.5 μm high, consisting of isolated conical warts, sometimes connected by very faint and fine lines; plage inamyloid, rarely centrally amyloid. Basidia 45.5–*63.5*–81.5(− 83) × 10–*13*–16 μm, subclavate, 4-spored. *Pleuromacrocystidia* (45–)46–*70.5*–95.5(− 103) × (4–)4.5–*6.5*–9(− 10) μm, abundant, cylindrical with tapering and fusiform apex, thin-walled. *Pleuropseudocystidia* 2.5–7 μm diam, not to slightly emergent. *Sterile elements* 37–*49*–62(64) × 3–*5*–7 μm, cylindrical, septate, with rounded apex. Lamellar edge sterile; marginal cells 13–*23.5*–34 × (5–)5.5–*9.5*–13(− 13.5) μm, cylindrical to clavate, sometimes septate. Hymenophoral trama mixed, with hyphae, lactifers and sphaerocytes. *Pileipellis* an irregular cutis, to 100 μm thick, consisting of loosely interwoven hyphae which are often slightly thick-walled and refringent, about 5–8 μm diam, but locally also swollen up to 20(− 30) μm; terminal hyphae with remarkable bulges and branchings often present. *Stipitipellis* a thin lamprotrichoderm on top of a layer of globose cells; all terminal elements refringent to slightly thick-walled, but some very thick-walled, 15–100 × 5–8 μm, cylindrical, with rounded or slightly tapering top; terminal elements anticline to very oblique, sometimes rather periclinally arranged.

*Ecology*: Found on soil in montane forests with *Quercus humboldtii*, *Q. seemannii*, *Q. copeyensis,* and *Quercus* sp.

*Distribution*: Known from Costa Rica, Panama, and Colombia.

*Remarks*: As *Lactifluus domingensis, L. hallingii* bears great resemblance to some of the other species from the *L. deceptivus* species complex, both macro- and microscopically. In the phylogeny (Fig. 1), we find that *L. hallingii* is most closely related to a clade with a North American distribution: clade 2 (Fig. 1). Clade 2 differs from *L. hallingii* in the smaller basidia (30.5–*42.5*–54 × 7.5–*11.5*–16 μm) and the shorter macrocystidia (29–*52*–75 × 5–*9*–13.5 μm). Compared to *L. hallingii*, the species described from Florida are relatively easily distinguishable: *L. arcuatus* has distinctly smaller spores (4–6 μm long), and *L. caeruleitinctus* has blue tinges in the stipe which are lacking in *L. hallingii*. *Lactifluus domingensis* shares the Neotropical distribution, but that species was found with the endemic *Pinus occidentalis* on the island of Hispaniola. For microscopic differences between these two species, see the remarks under *L. domingensis*.

*Other specimens examined:* **Colombia**: *Antioquia*: Municipio Santa Rosa de Osos, near Llanos de Cuiva (6.75°; − 75.5°, alt. 2500 m), Andean forest, 5 Nov. 1986, *R. E. Halling 4977* (NY); vereda El Chaquiro, finca La Española (alt. 2700 m), with *Quercus humboldtii*, 12 June 1990, *A. E. Franco-Molano 523* (NY); Municipio de San Pedro, vereda La Pulgarina, with *Quercus humboltii*, 27 Apr. 1991, *A. E. Franco-Molano 555* (NY); Municipio Santa Rosa de Osos, corregimiento de Aragon, vereda El Quince, Finca San Bernardo (alt. 2900 m), 17 June 1991, *A. E. Franco-Molano 756* (NY)/ **– Costa Rica**: San José, Canton Dota, Jardin, 3.5 km W of Empalme (9.7144°; − 83.9744°, alt. 2220 m), with *Quercus seemannii* and *Q. copeyensis*, 07 Aug. 2000, *R. E. Halling 7993* (NY).

**Lactifluus arcuatus** (Murrill) Delgat, **comb. nov**.

MycoBank MB831087

*Basionym*: *Lactarius arcuatus* Murrill, *Mycologia* **33**: 440 (1941).

*Synonym*: *Lactaria arcuata* Murrill, *Mycologia* **33**: 440 (1941); orth. Var.

*Remarks*: Based on the original description, this species could fit morphologically in *L.* sect. *Albati* because of the white basidiome, the cottony inrolled margin, and the acrid latex. The placement in this section is confirmed molecularly by the position of the type sequence in the phylogeny (Fig. 1).

**Lactifluus caeruleitinctus** (Murrill) Delgat, **comb. nov**.

MycoBank MB831088

*Basionym*: *Lactarius caeruleitinctus* Murrill, *J. Elisha Mitchell Sci. Soc.* **55**: 368 (1939).

*Synonym*: *Lactaria caeruleitincta* Murrill, *J. Elisha Mitchell Sci. Soc.* **55**: 368 (1939); orth. Var.

*Remarks*: Based on Murrill’s notes on the type specimen, this species could fit morphologically in *L.* sect. *Albati* because of the white basidiome, the acrid taste, and the strongly unpleasant smell while drying. The placement of a collection identified as *L. caeruleitinctus* in *L.* sect. *Albati* (Fig. 1) further supports the placement of this species in *L.* sect. *Albati*.

**Lactifluus mordax** (Thiers) Delgat, **comb. nov**.

MycoBank MB832122

*Basionym*: *Lactarius mordax* Thiers, *Mycologia* **49**: 710 (1957).

*Remarks*: Based on the original description, this species could fit morphologically in *L.* sect. *Albati* because of the matted-tomentose margin, the very acrid latex, and the cuticular structure of the pileipellis. The placement in this section is confirmed molecularly by the position of the type sequence in the phylogeny (Fig. 1).

## DISCUSSION

A first attempt to find out which clade represents *L. deceptivus* was made by sequencing several collections from the studied material from Hesler & Smith’s description (Hesler and Smith 1979): collections *Schaffer 5895*, *Ammirati 2392*, *Smith 84,511,* and *Weber 4277*. However, our analysis inferred that these collections represent three different species: clades 2, 3, and 5 (Fig. 1), thus the interpretation of *L. deceptivus* remained elusive. In the phylogeny, there are several clades that have a relatively close distribution to where *L. deceptivus* was described (i.e. New York State): clades 2, 3, 5, and 12. The only microscopic character mentioned in the original description (Peck 1885) is the length of the spores (8.9–12.7 μm), so spores were measured for these four clades. Clade 12 has significantly smaller spores (5.9–*7*–8 × 4.6–*5.3*–6.1 μm), but the other three clades have similar spore sizes (clade 2: 9.6–*10.6–10.9*–11.9 × 7.5–*8.4–8.5*–9.5 μm; clade 3: 9.4–*10.6*–11.8 × 7.8–*8.5*–9.3 μm; clade 5: 9.9–*10.7–10.8*–11.9 × 7.4–*8*–8.6 μm), so all three clades were considered possible candidates to represent the true *L. deceptivus*.

*Lactifluus deceptivus* was described by Peck (1885), and Hesler and Smith (1979) designated a lectotype (Peck s.n., NYS-F-000959). Samples this old have both time and conservation related DNA damage, besides exogenous DNA contamination, that makes nucleic acid extraction and amplification challenging (Forin et al. 2018). Therefore, Illumina Miseq sequencing was chosen as an alternative to the conventional Sanger sequencing to overcome these problems for the type specimen of *L. deceptivus*. As expected, due to both the old age and the lack of precautions during the manipulation of specimens throughout the herbarium’s long life, contaminants are present in this specimen, and a total of 10 zero-radius ITS1 OTU’s were recovered from the sample. One *Lactifluus* sequence was picked up by the analysis. Phylogenetic analysis shows that this sequence belongs to clade 3, which was indeed considered a possible candidate based on distribution and spore measurements, revealing that this clade represents the true *L. deceptivus*.

Three other described species were found to belong in the complex: *L. arcuatus* and *L. caeruleitinctus*, described from Florida by Murrill (1939, 1941); and *L. mordax*, described from Texas by Thiers (Thiers 1957). Descriptions of Murrill’s species are rather concise, but both species have at least a clear character that sets them apart from *L. deceptivus*: *L. arcuatus* has distinctly smaller spores, and *L. caeruleitinctus* displays blue tinges in the stipe. *Lactifluus mordax* is can be macroscopically distinguished from *L. deceptivus* by the pileus colour, which is not white but yellow to cream, and microscopically by the smaller spores (7.5–9 × 6–7 μm) (Hesler and Smith 1979). A sequence was obtained for the holotypes of *L. arcuatus* and *L. mordax*, as well as a collection identified as *L. caeruleitinctus*, which shows that these species belong to a subclade (clades 9–15) of the *L. deceptivus* complex which is dominated by species known only from Florida (Fig. 1). Since *L. deceptivus* is situated in the other subclade (clades 1–8), these species are relatively more distantly related to *L. deceptivus*, which could explain why they are more easily distinguishable from it. *Lactifluus tomentoso-marginatus* was previously synonymised with *L. deceptivus* based on a detailed morphological study (Montoya and Bandala 2005), and the phylogeny confirms this synonymy by the position of the holotype in the same clade as the type of *L. deceptivus* (Fig. 1). However, other collections studied in the paper of Montoya and Bandala, originating from Mexico and initially identified as *L. tomentoso-marginatus*, were also considered to belong to the same species by the authors based on the morphological study. A sequence was obtained for one of these collections, and the phylogeny shows that it represents a distinct clade from *L. deceptivus* (clade 8, Fig. 1), further demonstrating the difficulty of morphologically delimiting species in this complex.

The *Lactifluus deceptivus* complex previously exclusively contained species described from the Nearctic. It was shown that species from this complex also occur in Indo-Malaya and the Neotropics, and two new Neotropical species are described. *Lactifluus domingensis* was found in the Dominican Republic, on the island of Hispaniola, while *L. hallingii* was found on the mainland, distributed across Costa Rica, Panama and Colombia. For most of the other clades, well-documented collections are lacking due to the previous perception that *L. deceptivus* represented just a single, easy to recognize species. In addition, many of the clades contain only one or two collections, so to further unravel this complex there is a need for more well-documented collections. Hopefully, *L. deceptivus s. lat*. Will be collected and described in more detail now that it is known to represent several morphologically similar species.

Since species in this complex resemble each other so strongly, they can be considered pseudocryptic species. This means that, while at first, they seem indistinguishable, they can be distinguished from each other once the appropriate character(s) is/are considered. This phenomenon is widespread in the genus *Lactifluus* (e.g. Stubbe et al. 2010; Van de Putte et al. 2010; Van de Putte 2012; De Crop et al. 2014; Van de Putte et al. 2016; Delgat et al. 2017; De Lange et al. 2018). For example, a very similar case to that described in this paper occurs in *L.* sect. *Lactifluus*: as with *L. deceptivus*, *L. volemus* was thought to be a single, easily recognizable species in Europe, with the same name also being applied on other continents. However, molecular analysis revealed a total of 35 species in this complex (Van de Putte 2012). Without molecular data it is next to impossible to delimit these pseudocryptic species, but once you ascertain which collections group together, morphological, although often subtle, differences may be found to distinguish between the species. Often, these species are relatively recently diverged from each other, as can be observed from the relatively short branch lengths (Fig. 1), and could explain why the morphologies have not diverged much from each other. Despite this limited morphological variability, we find that the *Lactifluus deceptivus* complex does contain a high diversity of species. The phylogeny (Fig. 1) reveals a total of at least 15 species, distributed across Asia, North and Central America.

Together with the clade around *Lactifluus vellereus*, another clade that can be considered a species complex, the *L. deceptivus* complex comprises *L.* sect. *Albati*. In contrast to species from the *L. deceptivus* clade, species from the *L. vellereus* clade have a lamprotrichoderm structure of the pileipellis with very long hairs (to 250–300(400) μm), causing the pileus surface to be extremely velutinous. The clade around *L. vellereus* contains at least 14 species (De Crop 2016; unpubl. results), which brings the total diversity of *L.* sect. *Albati* to 29 species. Considering that De Crop (2016) reports a total species diversity of 369 *Lactifluus* species, distributed across 37 clades, averaging ten species per clade, *L.* sect. *Albati* can be considered a relatively species-rich section. In addition, many of the species in the section are known from only one collection, so it can be expected that the diversity will be even higher.

It is noteworthy that the position of *L.* sect. *Albati* in *L.* subg. *Lactariopsis* is not supported in the phylogeny (Fig. 1). Also in the study of De Crop et al. 2017 this position was not supported in all separate gene phylogenies, which is why this section could be considered as a separate group from the subgenus. However, in order to favour more or less equal sized subgenera, (De Crop et al. (2017)) decided to include this section in *L.* subg. *Lactariopsis*. *Lactifluus* sect. *Albati* differs from the other sections in *L.* subg. *Lactariopsis* by the presence of macropleurocystidia, while true cystidia are lacking in most species of this subgenus. In addition, it is the only section in the subgenus which has temperate representatives.

Considering its name, it might be thought that *Lactarius pseudodeceptivus* would also belong to the clade around *L. deceptivus*. However, this species belongs in the genus *Lactarius,* as is confirmed by the placement of sequences of the holotype and paratypes in *Lactarius* (Fig. 1). There are some similarities with *L. deceptivus*, such as the inrolled cottony-tomentose margin and acrid taste. However, *Lactarius pseudodeceptivus* can be distinguished from species of the *Lactifluus deceptivus* complex, notably by the reticulated spore ornamentation and the ixocutis structure of the stipitipellis.

## CONCLUSION

*Lactifluus deceptivus* was previously thought to be a single, easily recognisable species. However, molecular analysis revealed that *Lactifluus deceptivus s. lat*. Represents a species complex containing at least 15 species, distributed across Asia and America. Despite the low morphological variability in this complex, it shows a relatively high species diversity. These species can be considered pseudocryptic species, meaning that (subtle) morphological differences may be found when studied in detail, as was done for two new Neotropical species: *Lactifluus hallingii* and *L. domingensis*. The identity of the true *L. deceptivus* is revealed. However, more well-documented collections are needed for most species in this complex, of which many are known from only one or two collections. Now that it was shown that *L. deceptivus s. lat*. Represents several morphologically similar species, the number of well-documented collections will hopefully rise substantially, ameliorating the possibility of fully resolving this species complex.

## Data Availability

All data generated or analysed during this study are included in this published article [and its supplementary information files].
